# 

**Published:** 1875-09

**Authors:** 


CONTENTS.

| | PAGE. |
|---|---|
| The Mystery of Dreams | 265 |
| Crazed by Tobacco | 268 |
| A Drugged Swimmer | 269 |
| To Save the Drowning | 270 |
| Ear-ache | 271 |
| A Remedial Institution | 272 |
| Our Summer at Maplewood—Chapter VIII.—Mrs. Rose's Omelet | 273 |
| Innoculation from Soap | 279 |
| Habits of Cleanliness | 280 |
| Health Food Company | 281,290 |
| Piles or Hemorrhoids | 282 |
| How Boys Earn Money | 283 |
| Healthy Eating | 284 |
| Bleeders | 285 |
| Indian Superstition | 286 |
| Privy Vaults | 286 |
| Suicides | 287 |
| Granulated Wheat Biscuit | 288 |
| Food for Teething Children | 289 |
| "Pomarius" | 289 |
| High-Pressure | 290 |
| Dinner or Lunch | 291 |
| Alcohol and Strychnine | 292 |
| Artificial Teeth | 293 |
| Drain and Air the Land | 294 |
| Electricity—Its Uses and Abuses | 295 |
| How Shall We Warm Our Houses? | 296 |
| Zero Refrigerator | 297 |
| Starvation vs. The "Ruling Passion." | 298 |
| Good Breeding | 299 |
| Darning By Machineey | 305 |

